# Annulus-guided Neochord Length Setting for Anterior Mitral Leaflet Repair

**DOI:** 10.1093/ejcts/ezag025

**Published:** 2026-01-16

**Authors:** Giuseppe Nasso, Alfredo Marchese, Giuseppe Santarpino, Raffaele Bonifazi, Tommaso Loizzo, Ernesto Greco, Flavio Fiore, Gaetano Contegiacomo, Giuseppe Speziale

**Affiliations:** Department of Cardiac Surgery, Anthea Hospital and Santa Maria Hospital, GVM Care & Research, Bari 70124, Italy; Department of Medicine and Surgery, LUM University, Casamassima, Bari 70124, Italy; Department of Cardiac Surgery, Anthea Hospital and Santa Maria Hospital, GVM Care & Research, Bari 70124, Italy; Department of Cardiac Surgery, Anthea Hospital and Santa Maria Hospital, GVM Care & Research, Bari 70124, Italy; Department of Cardiac Surgery, Anthea Hospital and Santa Maria Hospital, GVM Care & Research, Bari 70124, Italy; Department of Cardiac Surgery, Anthea Hospital and Santa Maria Hospital, GVM Care & Research, Bari 70124, Italy; Department of Health and Life Sciences, European University of Rome, 00163 Rome, Italy; Department of Cardiac Surgery, Anthea Hospital and Santa Maria Hospital, GVM Care & Research, Bari 70124, Italy; Department of Cardiac Surgery, Anthea Hospital and Santa Maria Hospital, GVM Care & Research, Bari 70124, Italy; Department of Cardiac Surgery, Anthea Hospital and Santa Maria Hospital, GVM Care & Research, Bari 70124, Italy; Department of Health and Life Sciences, European University of Rome, 00163 Rome, Italy

## Introduction

Accurate neochordal length is the cornerstone of durable anterior mitral leaflet repair.^1–3^ Visual estimation and hydrostatic testing are inherently load-dependent and vary among surgeons, exposing patients to the risks of residual prolapse, leaflet restriction with elevated transmitral gradients, or systolic anterior motion (SAM). To remove these sources of error, we standardized an annulus-referenced method with a temporary annular-guided chordal sizing technique (TRACK). Each expanded polytetrafluoroethylene (ePTFE) neochord is tied at a constant height aligned with the native annular plane.

The clinical goal is a broad, posteriorized, SAM-sparing coaptation band, aiming for generous leaflet coaptation (typically around ≥10 mm at A2-P2), in line with data linking greater coaptation length to repair durability.^4^ In this report, we present a step-by-step description of the current sequence and summarize results from a consecutive series. This retrospective observational study was conducted at a single high-volume mitral centre, Anthea Hospital—GVM Care & Research—and adheres to the STROBE guidelines for observational studies. The study protocol was approved (May 2, 2025) by our Institutional Review Board (IRB No. 6.2025). Written informed consent for the collection and use of anonymized clinical and echocardiographic data for research and publication was obtained from all patients in accordance with institutional protocol.

## Technique

Candidates are adults with degenerative mitral regurgitation (MR) and anterior leaflet prolapse or flail suitable for repair via right anterolateral minithoracotomy or sternotomy.

### Papillary anchoring

**Figure 1.**
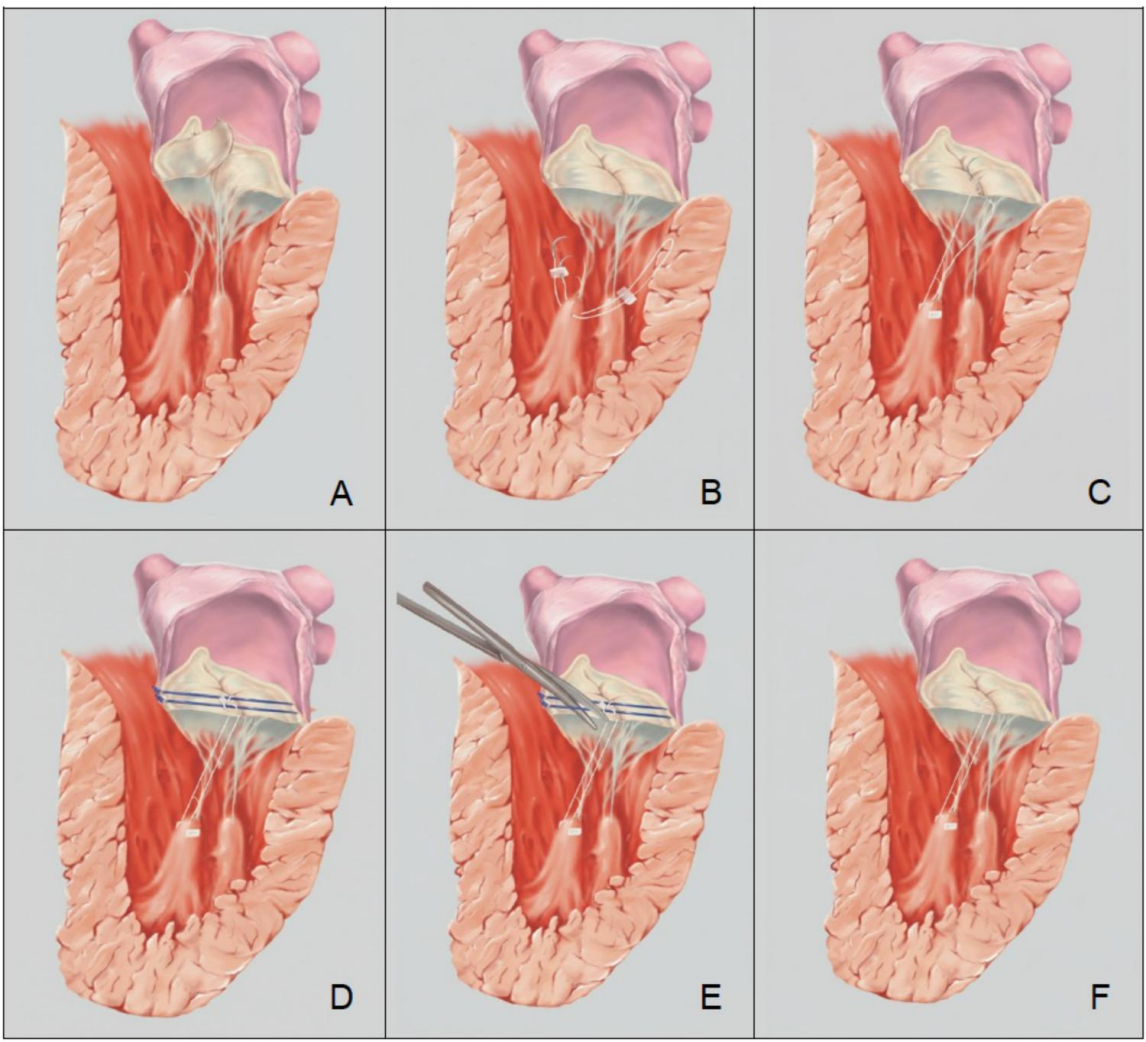
Stepwise Annular-guided Neochordae Adjustment and Final Broad Leaflet Coaptation.

After detecting flail or prolapse of the anterior leaflet scallop, each ePTFE neochord is anchored to the appropriate papillary muscle head (anterolateral or posteromedial) with deep bites into fibrous tissue and secure knots. The line of pull should reproduce the native chordal vector towards the intended anterior segment to avoid eccentric tension. For isolated A2 disease, 1-2 neochord pairs usually suffice; extension to A1 or A3 is added when prolapse is more diffuse, maintaining symmetry (**Figure 1A and B**).

### Leaflet passage

The 2 limbs of the ePTFE suture are passed twice through the free edge of the prolapsing anterior leaflet from the ventricular to the atrial side at a distance of approximately 5 mm, creating 2 loops that are left untied (**Figure 1C**).

### Temporary annular guide

A dedicated temporary guide is then positioned flush on the true annular plane at the target anterior scallop with prolapse or flail (typically A2). The guide must be strictly coplanar with the annulus; even minor tilt will distort measured height. In detail, a single 2-0 braided polyester suture (Ethibond Excel, Ethicon Inc.) is passed through the anterior annulus at a location corresponding to the diseased leaflet segment. The Ethibond needle tips are then individually slipped through the 2 loops of the previously implanted artificial neochord and passed through the posterior annulus of the opposing segment, at a distance of a few millimeters from one another. The Ethibond stitch is kept under tension in order to create a temporary guide, like a track, for chordal attachment. The 2 free ends of each neochord are then adjusted to the level defined by the temporary Ethibond suture height and tied right above it. Finally, the temporary Ethibond “track” is cut and removed. The guide functions as an intracardiac calliper, providing a fixed reference that is independent of ventricular filling. Because height is set against the annular plane rather than against ventricular pressurization, the measurement is insensitive to saline load and haemodynamic variation. This temporary lock prevents creep during definitive knot tying (**Figure 1D-F**).

A complete semi-rigid ring is implanted as the last step; however, the choice of the annuloplasty device reflects personal surgeon preference, and the same technique is conceptually applicable to any type of ring or band.

A real-time surgical demonstration of annulus-guided neochord length setting (TRACK) is provided in **Video 1**.

## Results

From January 2020 to October 2024, 60 adults underwent TRACK repair; 54 (90%) via minithoracotomy and 6 via sternotomy for concomitant procedures. Technical success was 100% (intraoperative MR ≤ mild with broad posteriorized coaptation and no SAM). Thirty-day outcomes were: mortality 0%, de-novo atrial fibrillation 20%, re-exploration for bleeding 1.7%, and mechanical ventilation >24 hours 1.7%, with no stroke or dialysis-requiring acute kidney injury. Follow-up was complete in all patients (mean 24 ± 12, median 22 [12-36] months; data-lock October 31, 2025); survival was 100%, with 1 mitral valve replacement for infective endocarditis. At last echocardiographic assessment of native valves (*n* = 59), MR was ≤1+ in all, mean transmitral gradient 3.0 ± 1.1 mmHg, coaptation length 11.0 ± 1.3 mm, no SAM, left-ventricular ejection fraction 56 ± 7%, and 88% in NYHA class I. Detailed baseline, operative, and follow-up data are summarized in **Table 1**.

**Table 1.** Baseline characteristics, procedural details, and follow-up outcomes of the study cohort (n = 60)

Preoperative	Value (%)
Age (years)	67 ± 11
Male	38 (63)
Hypertension	47 (78)
Hypercholesterolaemia	33 (55)
Diabetes mellitus	22 (37)
COPD	7 (12)
Renal dysfunction	10 (17)
Preoperative atrial fibrillation	20 (33)
LVEF (%)	54 ± 8
EuroSCORE II (%)	5.2 ± 2.1
Anterior leaflet disease only	36 (60)
**Intraoperative**	
Minithoracotomy/Sternotomy^a^	54 (90)/6 (10)
Concomitant tricuspid annuloplasty	13/54 (24)
CPB (min)	92 ± 35
Aortic cross-clamp time (min)	55 ± 22
ePTFE neochords, median	2 [1-4]
Complete ring size (mm), median	34 [32-38]
Concomitant CABG and/or AVR^a^	6 (10)
Conversion to sternotomy	0
Technical success (MR ≤ mild, no SAM)	60 (100)
**30-day outcomes**	
Mortality	0
Re-exploration for bleeding	1 (1.7)
Prolonged ventilation >24 h	1 (1.7)
Stroke	0
dialysis	0
De novo atrial fibrillation	12 (20)
wound complications	0
Clinically relevant SAM/LVOT obstruction	0
ICU length of stay (days)	2.9 ± 1.0
Total hospital length of stay (days)	8.6 ± 2.5
**Follow-up**	
Completeness	60/60 (100)
Follow-up (months), mean ± SD	**24 ± 12**
Follow-up (months), median [IQR]	22 [12-36]
Survival	100%
**Valve reintervention**	**1/60 (1.7%)**
MR at last echo^b^	None/trace or mild in **all native valves** (59/59, 100)
Mean transmitral gradient (mmHg)	3.0 ± 1.1
Coaptation length A2-P2 (mm)	11.0 ± 1.3
SAM/LVOT obstruction	0
LVEF (%)	56 ± 7
PASP (mmHg)	34 ± 8
NYHA class I/II	53 (88)/7 (12)

Format: *n* (%) or mean ± SD; if indicated: median [IQR]. Data-lock: October 31, 2025. Follow-up duration = time from index repair to last contact/echo per patient (arithmetic mean).

a Median sternotomy is the standard approach when concomitant procedures are required; minithoracotomy for isolated MV repair.

b Denominator 59: 1 patient underwent MVR for endocarditis (no native-valve echo at follow-up).

Abbreviations: AVR, aortic valve replacement; CABG, coronary artery bypass grafting; COPD, chronic obstructive pulmonary disease; CPB, cardiopulmonary bypass; EuroSCORE II, European System for Cardiac Operative Risk Evaluation II; ICU, intensive care unit; IQR, interquartile range; LVEF, left ventricular ejection fraction; LVOT, left ventricular outflow tract; NYHA, New York Heart Association; PASP, pulmonary artery systolic pressure; SD, standard deviation; ePTFE, expanded polytetrafluoroethylene; MR, mitral regurgitation; SAM, systolic anterior motion.

Boldface indicates key follow-up outcomes (survival, valve reintervention, and MR grade at last echocardiography).

## Discussion

Annulus-guided neochordal sizing directly addresses the main limitation of conventional artificial chordae techniques: the difficulty of setting an accurate and reproducible neochord height.^1–3^ The TRACK concept uses the mitral annulus itself as intracardiac reference, allowing neochord to be tied at a constant annulus-to-edge distance.

A second key is the explicit focus on coaptation geometry rather than on lesion type alone. By focusing on generous coaptation at A2-P2 (with a clinical target around ≥10 mm when feasible), TRACK operationalizes the growing evidence that greater coaptation length predicts durability after degenerative mitral repair.^4^

In this consecutive series, the annulus-guided sequence yielded uniform intraoperative competence, with a minimally invasive setting.

Track-specific contribution is to translate the annulus-guided concept into a detailed, reproducible operative protocol. Whereas our previous comparative study focused on long-term outcomes of TRACK versus conventional artificial chordae sizing,^5^ the present article concentrates on indications, instrumentation, order of steps, and practical safeguards for anterior leaflet repair. In this sense, TRACK can be viewed as a modular, anatomy-based sizing strategy that can be integrated with different posterior repair techniques and access routes, rather than as a competing leaflet-reconstruction philosophy.

## Data Availability

All data relevant to this study are included in the article. De-identified individual patient data and additional material that support the findings of this study are available from the corresponding author.
